# Inhibitory effects of low intensity pulsed ultrasound on osteoclastogenesis induced in vitro by breast cancer cells

**DOI:** 10.1186/s13046-018-0868-2

**Published:** 2018-08-20

**Authors:** Valeria Carina, Viviana Costa, Stefania Pagani, Angela De Luca, Lavinia Raimondi, Daniele Bellavia, Stefania Setti, Milena Fini, Gianluca Giavaresi

**Affiliations:** 1 IRCCS Rizzoli Orthopedic Institute, Bologna, Italy; 2 Laboratory of Preclinical and Surgical Studies, IRCCS Rizzoli Orthopedic Institute, Bologna, Italy; 3 IGEA SpA, Clinical Biophysics, Carpi, Modena Italy

**Keywords:** Osteolytic metastasis, Low intensity pulsed ultrasound, Osteoclasts, Breast cancer

## Abstract

**Background:**

Bone tissue is one of the main sites for breast metastasis; patients diagnosed with advanced breast cancer mostly develop bone metastasis characterized by severe osteolytic lesions, which heavily influence their life quality. Low Intensity Pulsed Ultrasound (LIPUS) is a form of mechanical energy able to modulate various molecular pathways both in cancer and in health cells.

The purpose of the present study was to evaluate for the first time, the ability of LIPUS to modulate osteolytic capability of breast cancer cells.

**Methods:**

Two different approaches were employed: a) Indirect method -conditioned medium obtained by MDA-MB-231 cell line treated or untreated with LIPUS was used to induce osteoclast differentiation of murine macrophage Raw264.7 cell line; and b) Direct method -MDA-MB-231 were co-cultured with Raw264.7 cells and treated or untreated with LIPUS.

**Results:**

LIPUS treatment impaired MDA-MB-231 cell dependentosteoclast differentiation and produced a reduction of osteoclast markers such as Cathepsin K, Matrix Metalloproteinase 9 and Tartrate Resistant Acid Phosphatase, suggesting its role as an effective and safe adjuvant in bone metastasis management.

**Conclusion:**

LIPUS treatment could be a good and safety therapeutic adjuvant in osteolyitic bone metastasis not only for the induction properties of bone regeneration, but also for the reduction of osteolysis.

## Background

Breast cancer is one of the most common tumors affecting women [1, 2]. Although the prognosis of breast cancer patients is generally favorable due to early diagnosis and advances in therapies, 20–30% of patients will develop distant metastases that drastically reduce their survival time [3, 4]. The most common sites for breast cancer metastasis are bone, liver, lung, brain and skin, and these are associated with the patients’ survival outcome [5, 6]. Bone tissue is indeed a preferential site of metastatic breast cancer cells, which act enhancing mostly bone resorption and inhibiting bone formation. This unbalance in bone remodeling leads to skeletal complications related to the affected bone segment [7] including, severe pain, bone instability, fractures, spinal cord compression, hypercalcaemia and bone marrow aplasia [8], which rapidly deteriorate patients quality of life.

Metastatic breast cancer cells act releasing bone resorption factors that stimulate osteoclastic differentiation directly [9, 10] or indirectly acting through osteoblasts [11]. In particular, metastatic breast cancer cells promote osteoclasts formation and activity by secreting osteolytic interleukins (ILs) such as IL-8 and IL-11,as well as TNFαand matrix metalloproteinases (MMP1, MMP2),which are involved in bone matrix destruction [12]. Moreover, metastatic breast cancer cells express factors, such as parathyroid hormone-related peptide (PTHrP) that induces osteoblasts to produce mediators (RANKL, PGE, IL-11), which subsequently stimulate osteoclast differentiation from monocyte precursors [11, 13].

Strategies to treat bone metastases depend on the number of metastases, their location and prognosis. A radiotherapeutic or systemic approach is preferred in case of multiple metastases or lesions sensitive to adjuvant therapies. If metastatic lesions are unresponsive to chemotherapy, but their localization allows surgery and the patient presents good clinical conditions, it is preferable to treat metastases surgically [14, 15]. If surgery is not recommended, or if the prognosis is unfavorable, patients should undergo palliative care with the primary aim of alleviating debilitating pain and improving their quality of life. Bone pain is usually managed via a multimodality approach, including the use of analgesic medications, cytotoxic chemotherapy, hormone-deprivation therapy, radiation therapy as well as administration of bisphosphonates, bone-seeking radiopharmaceuticals [16–18] or minimally invasive percutaneous therapies such as radiofrequency, thermoablation with microwave [19, 20], cryoablation [21], alcholization [22], electrochemotherapy [23] or high intensity focused ultrasounds (HIFU) [24].

Recently, the therapeutic potential of ultrasound in pain reduction and oncology has been evaluated. The possibility of using ultrasounds with different parameters: intensity (Low < 3 W/cm^2^; High ≥3 W/cm^2^) and frequency (Low 20–200 kHz; High 1–20 MHz) [25] allows their application in various fields, ranging from tissue ablation (using high intensity focused ultrasound-HIFU) [26, 27] to tissue regeneration (using low intensity ultrasound) [28, 29].

Nowadays, HIFU, or focused ultrasound (FUS), is used in oncological therapy not only to ablate metastasis, but also to relief pain [24, 30]. Like HIFU, low intensity pulsed ultrasound (LIPUS) is transmitted into tissues as an acoustic pressure wave, leading downstream effects by translating this mechanical signal into a biochemical response via integrin mechano-receptors. Mechanical forces (mechanotransduction), either generated by cell contractions or from external sources, have been demonstrated to have strong effects on cell differentiation, growth, and survival [31]; [32]. Through mechanotransduction mechanisms, LIPUS is able to trigger alterations in gene expression. The most studied pathways modulated by LIPUS concern regeneration of bone tissue and acceleration of bone repair processes by up-regulating bone specific genes and inducing osteoblast differentiation [33, 34]. In addition it is known that LIPUS is able to modulate gene expression and release soluble factors involved in inflammatory and membrane degradative processes such as ILs, MMPs and MAPKs in both healthy and cancer cells [33, 35–37].

Starting from these knowledges on osteoblasts and cancer cells, the aim of the present study was to evaluate the effect of LIPUS on the osteoclastic differentiation process induced by breast cancer cells. Within bone metastatic microenvironment induced by breast cancer cells, different cell types coexist such as healthy bone cells, osteoblasts and osteoclasts, which are continually stimulated by both cancer cells and osteolytic processes. By considering the modulating effect of LIPUS on bone cells, we hypothesized, for the first time, that LIPUS could modulate the release of mediators from breast cancer cells, which could be able to act on osteoclast, thus reducing bone resorption and preventing metastatic osteolytic progression. In this sense, this study allowed to achieve the first information for the development of a new therapeutic approach for metastasis treatment, where LIPUS become an adjuvant of the current pharmacological therapy. In this regard we employed murine macrophage cells (Raw264.7) that are a widely used system to analyze osteoclastic differentiation [38, 39] and MDA-MB-231,a metastatic breast cancer cell line that is largely employed to study osteoclastic differentiation in co-culture [40] or conditioned medium systems [38]. Two different culture approaches were applied: (a) indirect method where conditioned medium obtained by the breast cancer cell line MDA-MB-231 treated or untreated by LIPUS was used to induce osteoclast differentiation of murine macrophage Raw264.7 cell line; (b) direct method where MDA-MB-231 cells were co-cultured with Raw264.7 cells and treated or untreated with LIPUS.

## Methods

### Cell lines

The breast cancer cell line MDA-MB-231 (HTB-26™), purchased from ATCC®, was cultured at 37 °C and 5% CO_2_ in Dulbecco’s modified Eagle’s medium with high glucose (DMEM) (Euroclone S.p.A., Pero, Milano, Italy) supplemented with 10% heat-inactivated fetal bovine serum (FBS,) (Lonza, Verviers, Belgium), 1 mM Sodium Pyruvate (Euroclone), 2 mM glutamine, 100 U/ml penicillin and 100 μg/ml streptomycin (Gibco, Invitrogen Corp., Carlsbad, CA, USA). Murine macrophage Raw264.7 cells were purchased from ATCC® and cultured at 37 °C and 5% CO_2_in DMEM supplemented with 10% FBS, 2 mM glutamine and antibiotics (100 U/ml penicillin, 100 μg/ml streptomycin).

### Conditioned media preparation

**Fig. 1:**
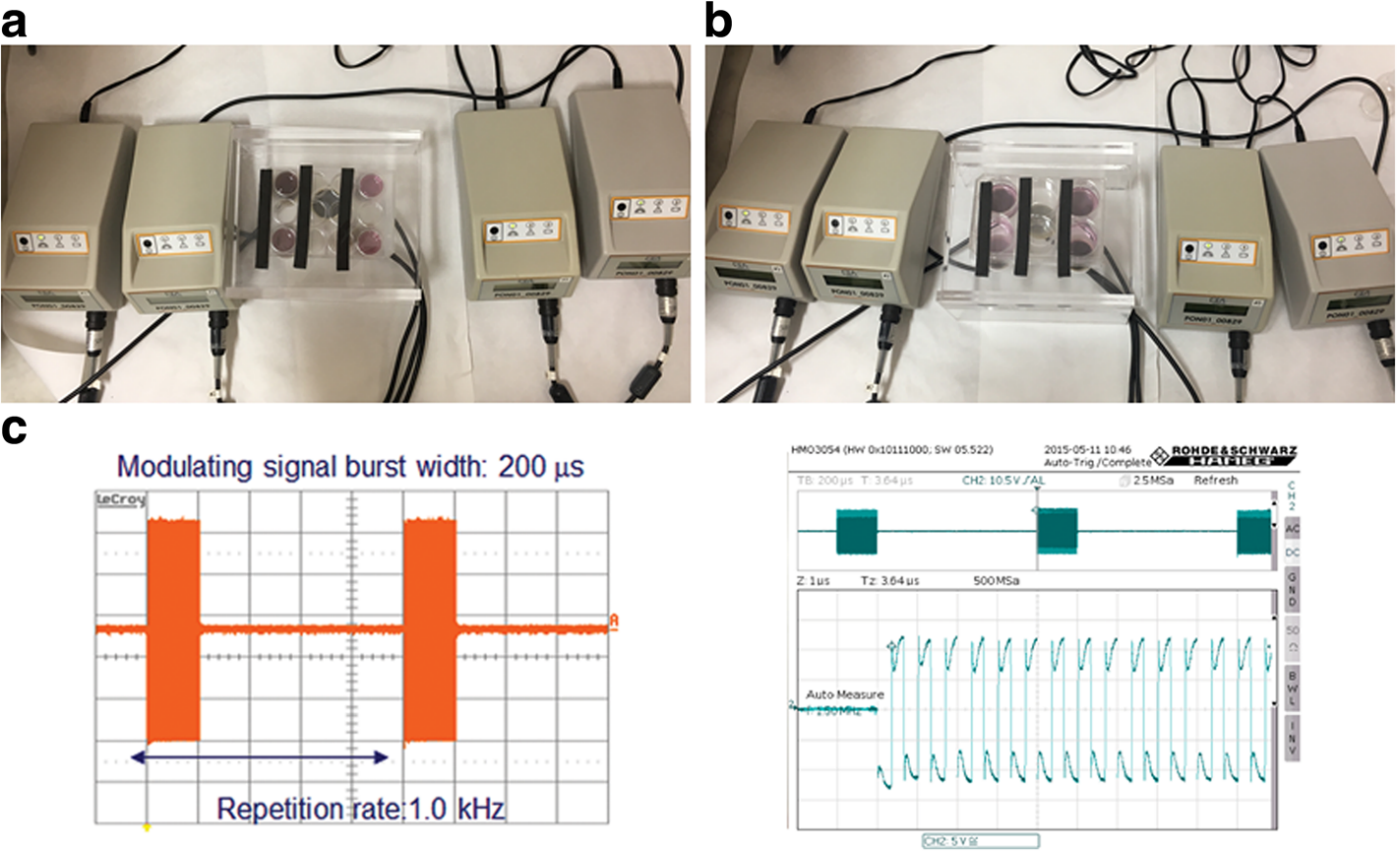
LIPUS set-up experiment. Upper panel: LIPUS transducer device. Bottom panel: characteristic of the ultrasound signal: 200 μs burst of 1.5 MHz sine waves repeated at 1 kHz (**c**). MDA-MB-231 cells were seeded in 12-well plates employing four wells/plate. Two wells were placed on swich-on transducer receiving LIPUS stimuli to obtain LCM the other two were placed on swich-off transducer and used as untreated group to collect CM. LIPUS treatment was performed for 20 min/day for 10 days (**a**). MDA-MB-231 cells were seeded in 6-well plates using four wells/plate. After 24 h, a transwell insert was inserted into each well and Raw264.7 cells were seeded in the upper compartment. Two wells were placed on swich-on transducer receiving LIPUS stimuli to obtain LIPUS_CC group, the other two were placed on swich-off transducer and used as untreated to obtain Untreated_CC group. LIPUS treatment was performed for 20 min/day for 10 days (**b**)

MDA-MB-231 cells were seeded in 12-well plates at a density of 20,000 cells/well to prepare different conditioned media for subsequent studies and then divided in two groups. A group of MDA-MB-231 cells was stimulated with LIPUS 20 min/day for 10 days (Fig. 1a). The medium was changed and discarded at day 3 and 6, then for the medium of the final 4 days LIPUS conditioned medium (LCM) was collected, filtered (0.22 μm), aliquoted and frozen at − 80 °C. The other group of MDA-MB-231 cells was maintained at the same conditions of the previous group but without LIPUS stimulation, then conditioned medium (CM) was harvested and stored as described above.

### Raw264.7 osteoclastic differentiation

Raw264.7 cells were seeded in 24-well plates at a density of 5000 or 2500 cells/well and treated (for 6 days for gene expression analysis and for 10 days for protein release evaluation) with 10% of conditioned medium derived by untreated (CM) or LIPUS treated (LCM) MDA-MB-231. Alternatively, as positive control, Raw264.7 were seeded at the same density and treated with 25 ng/ml human recombinant RANK Ligand (Gibco, Life Technologies, USA) for 6 or 10 days for gene expression analysis and for protein release evaluation, respectively. The medium was changed every three days.

### Co-cultures

MDA-MB-231 cells were seeded in 6-well plates at a density of 30,000 cells/well. After 24 h, a transwell insert (pore size 0.4 μm) (Millipore, Cork, Ireland) was inserted into each well and Raw264.7 cells were seeded in the upper compartment at a density of 15,000 cells/well. Then, co-cultures were divided in two groups: a group stimulated with LIPUS 20 min/day for 10 days (LIPUS_CC); and the other group maintained at the same conditions of the previous group but without LIPUS stimulation (Untreated_CC) (Fig. 1b).

### LIPUS treatment

The LIPUS exposure device was manufactured by IGEA SpA (Carpi-Modena, Italy). It consisted of an array of 5 autonomous transducers, designed for use in a multi-well culture plate. LIPUS signal consisted of 200 μs burst of 1.5 MHz sine waves, repeating at 1 kHz and delivering 30 mW/cm^2^SATA intensity, transmitted through the bottom of the culture dish via the coupling gel between the ultrasonic transducer and the dish (Fig. 1a-c). A calibrated force balance measured the power of the collimated ultrasound beam emitted from the transducer (Ultrasound Power Meters UPM-DT-1AV, Ohmic Instruments, St. Charles – MI, US). The mediated power was 33.7 mW/cm^2^ [33, 41].

### MDA-MB-231 cell viability

WST-1 colorimetric reagent (Roche Diagnostics GmbH, Manheim, Germany) was used to evaluate MDA-MB-231cell viability after 10 days of LIPUS treatment. Briefly, WST-1 reagent (10% vol/vol) was added to the cell monolayer in each well. After 4 h of incubation, formazan dye produced by viable cells was quantified spectrophotometrically at 450 nm by Bio-Rad Microplate Reader (Bio-Rad Laboratories, Hercules, CA, USA) and results were reported as percentage of viable cells compared to untreated LIPUS MDA-MB-231.

### TRAP staining assay

Raw264.7 cells were cultured as described above (Raw264.7 osteoclastic differentiation and co-culture paragraphs) and stained for detection of tartrate resistant acid phosphatase (TRAP) activity, according to the manufacturer’s protocol (Acid Phosphatase, Leukocyte TRAP Kit; Sigma–Aldrich, St. Louis, MO, USA) and evaluated by light microscopy (Eclipse Ti-S, Nikon). Multinucleated TRAP^+^ cells containing more than three nuclei were scored as mature osteoclasts. TRAP^+^ cells were counted for each condition from three different fields in three independent experiment.

### qRT-PCR analysis

Total RNA from Raw264.7 cells treated as described above (Raw264.7 osteoclastic differentiation and co-culture paragraphs) was extracted using a PureLink™ RNA Micro Kit (Invitrogen™) and reverse-transcribed with a High Capacity cDNA Reverse Transcription Kit (Applied Biosystems™, Life Technologies - Italy) following the manufacturer’s instructions. Each cDNA sample was tested in duplicate. qRT-PCR analysis was performed by using the SYBR® Green Real-Time PCR Master Mix (Applied Biosisystems™). Custom made primers (Invitrogen™) employed are reported in Table 1. The mean threshold cycle was used for the calculation of relative expression using the 2^-ΔΔCt^ method, against *Gapdh* as housekeeping gene and Raw264.7 control cultures (CTR – maintained in their growth medium) as calibrator [42].

**Table 1.** Initrogen^TM^ sequences employed for gene expression studies

Gene	Forward primer sequence (5′-3′)	Reverse primer sequence (3′-5′)
Gapdh	CCCAGAAGACTGTGGATGG	CAGATTGGGGGTAGGAACAC
Acp5	GCGACCATTGTTAGCCACATACG	CGTTGATGTCGCACAGAGGGAT
Ctsk	GCGTTGTTCTTATTCCGAGC	CAGCAGAGGTGTGTACTATG
Mmp9	GCTGACTACGATAAGGACGGCA	GCGGCCCTCAAAGATGAACGG

### ELISA assay

Raw264.7 cells were treated as described above (Raw264.7 osteoclastic differentiation and co-culture paragraphs) and MMP9 (Matrix Metalloproteinase 9) and CTSK (Cathepsin K) secreted by Raw264.7 cells were quantified respectively by mouse MMP9 ELISA assays and CTSK ELISA assay (Wuhan Fine Biological Technology Co., Ltd) according to the manufacturer’s instructions and normalized vs. dsDNA content calculated by using fluorimetric Quant-iTPicoGreen dsDNA Assay Kit (Invitrogen™, Life Technologies - EuroClone S.p.A, Pero-Milan, Italy).

### SEM analysis

To perform SEM analysis, Raw264.7 cells were seeded on glass coverslips and cultured as described above (Raw264.7 osteoclastic differentiation and co-culture paragraphs). Each coverslip was fixed at room temperature for 1 h in 2.5% glutaraldehyde in 0.1 M Phosphate buffer at pH 7.4. The fixed samples were dehydrated in graded series of ethanol (10, 20, 30, 50, 70, 90, 100% for 10 min each, with 3 changes at 100%), and finally twice in hexamethyldisilazane. After gold-sputtering (B7340 Manual Sputter Coater Assing SpA) samples were then examined by scanning electron microscopy (EVO LS - ZEISS, Assing SpA). Backscattered electron observations were performed at 20 kV.

### Statistical analysis

Statistical analysis was performed using R v.3.3.3 software [43]. Data were reported as bar chart or mean ± SD at a significant level of *p* < 0.05. After having verified normal distribution (Shapiro-Wilk test) and homogeneity of variance (Levene test), data were analyzed by means of Student *t* test by considering we hypothesized that: (1) the recombinant RANKL was able to induce osteoclastic differentiation in Raw264.7 cell line; (2) the conditioned medium of LIPUS treated MDA-MB-231 cells (LCM) was able to interfere on Raw264.7 cells osteoclastic differentiation compared to the conditioned medium of untreated ones (CM); (3) LIPUS treatment of co-cultured MDA-MB-231 and Raw264.7 cells (LIPUS_CC) was able to reduce osteoclastic differentiation in Raw264.7 cells compared to untreated co-cultures (Untreated_CC). The effect size of comparisons derived by these hypotheses was reported as standardized Cohen’s *d*, which is defined as the difference between two means divided by pooled standard deviation. For all the tests performed, 3 independent experiments were conducted in triplicate for each group.

## Results

### Cell viability

**Fig. 2:**
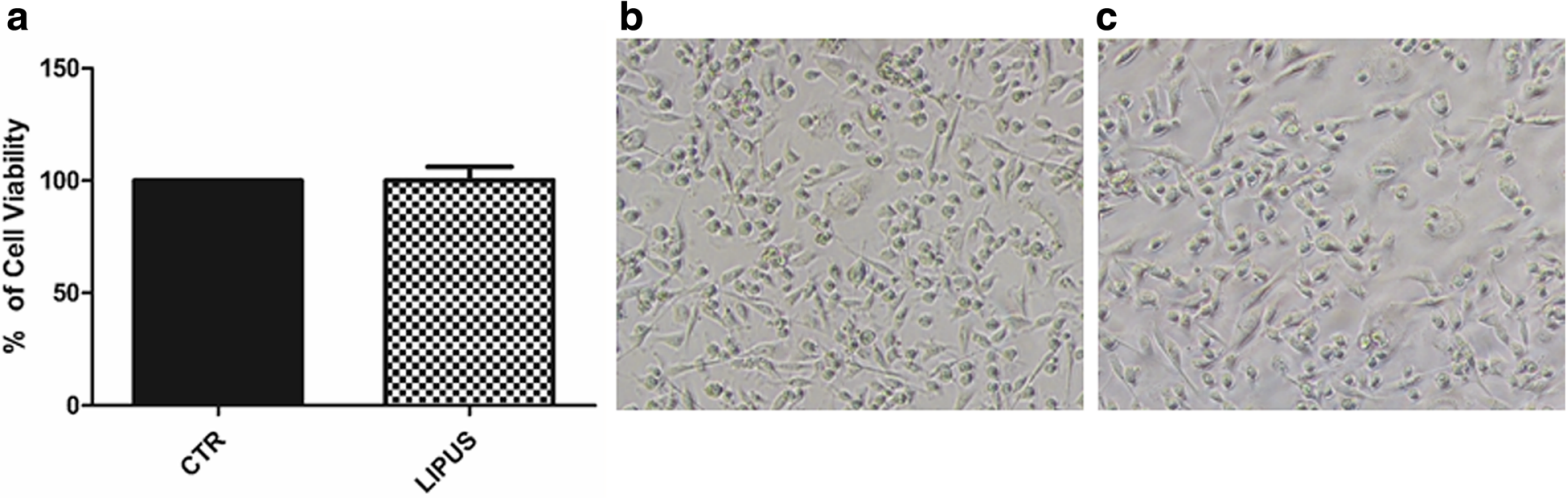
Effects of LIPUS treatment on MDA-MB-231 cells. MDA-MB-231 breast cancer cell line was cultured for 10 days and treated with LIPUS for 20 min/day or left untreated. Results of WST1 assay (**a**) expressed as percentage of untreated cells (100%) (Mean ± SD, *n* = 3 replicates). Cell morphology of MDA-MB-231 untreated (**b**) and treated (**c**) with LIPUS (20× magnification, Eclipse Ti-S, Nikon)

To evaluate the effects of LIPUS treatment on cancer cells viability, the LIPUS stimuli on MDA-MB-231 was measured. LIPUS stimulation for 10 days did not influence (*d* = 0.09, *p* = 0.987) viability of MDA-MB-231 cell culture, as shown by WST1 assay (Fig. 2a) and optical microscope analysis showed that it did not affect cell morphology either (Fig. 2b and c), demonstrating that treatment with LIPUS is usable in a tumor system.

### Osteoclast differentiation markers

**Fig. 3:**
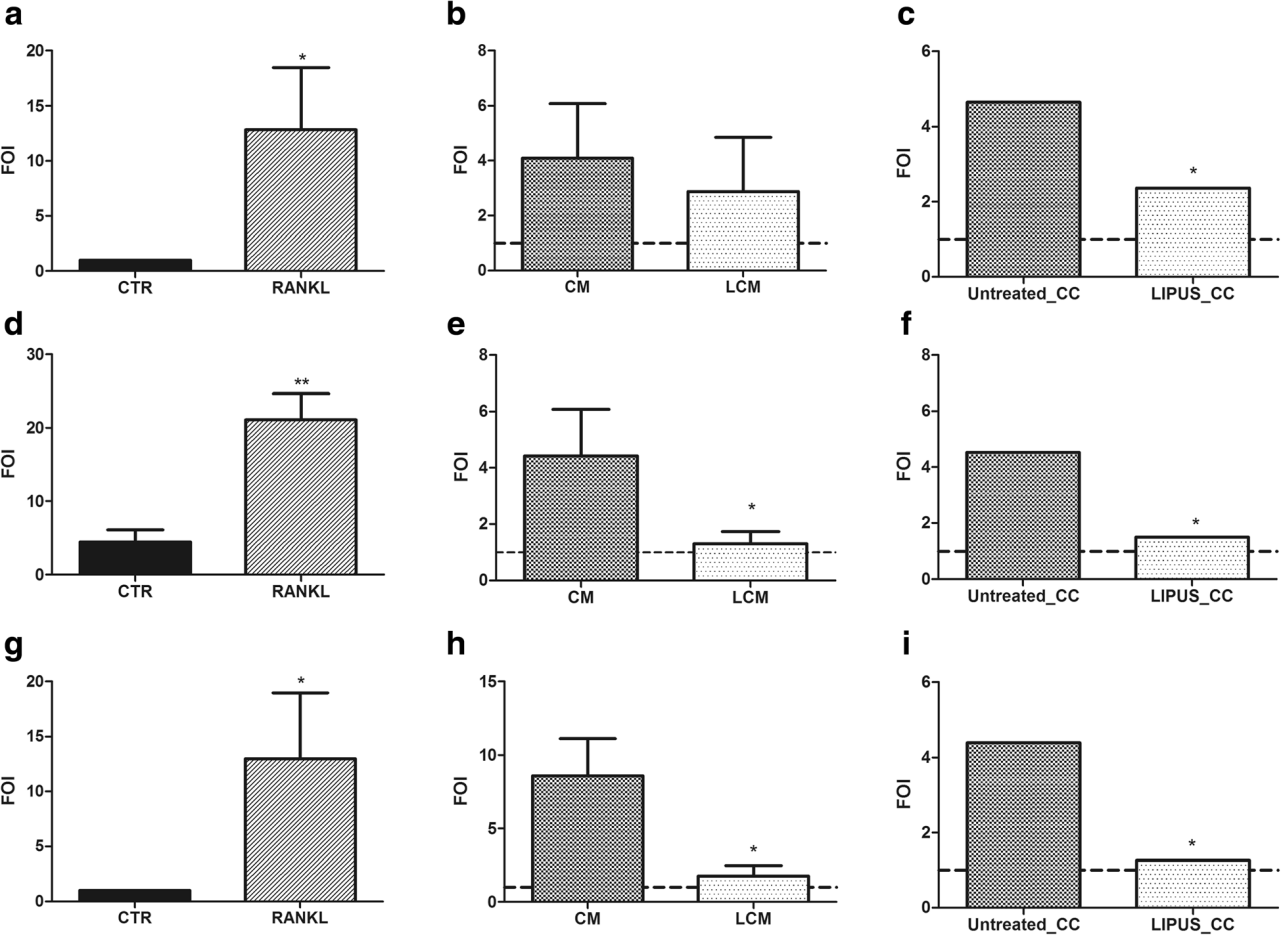
Gene expression analysis. Quantitative RT-PCR of *Trap* (**a**-**c**), *Ctsk* (**d**-**f**) and *Mmp9* (**g**-**i**) in Raw264.7 cells cultured with: (**a**, **d**, **g**) RANKL used as positive control; (**b**, **e**, **h**) conditioned medium harvested from untreated MDA-MB-231cell cultures (CM) or conditioned medium form cells treated with LIPUS (LCM); and (**c**, **f**, **i**) co-cultured with MDA-MB-231 cells untreated (Untreated_CC) or treated (LIPUS_CC) with LIPUS. PCR data were expressed as fold of change (2^-ΔΔCt^) vs. the calibrator (CTR) (Mean ± SD, *n* = 3 replicates). Student *t* test: *p < 0.05, **p < 0.005, ***p < 0.0005. Effect sizes between RANKL and CTR: *Trap* (**a**) *d* = 2.3, *p* = 0.022; *Ctsk* (**d**) *d* = − 8.0, *p* = 0.001; and *Mmp9* (**g**) *d* = − 2.0, *p* = 0.047

**Fig. 4:**
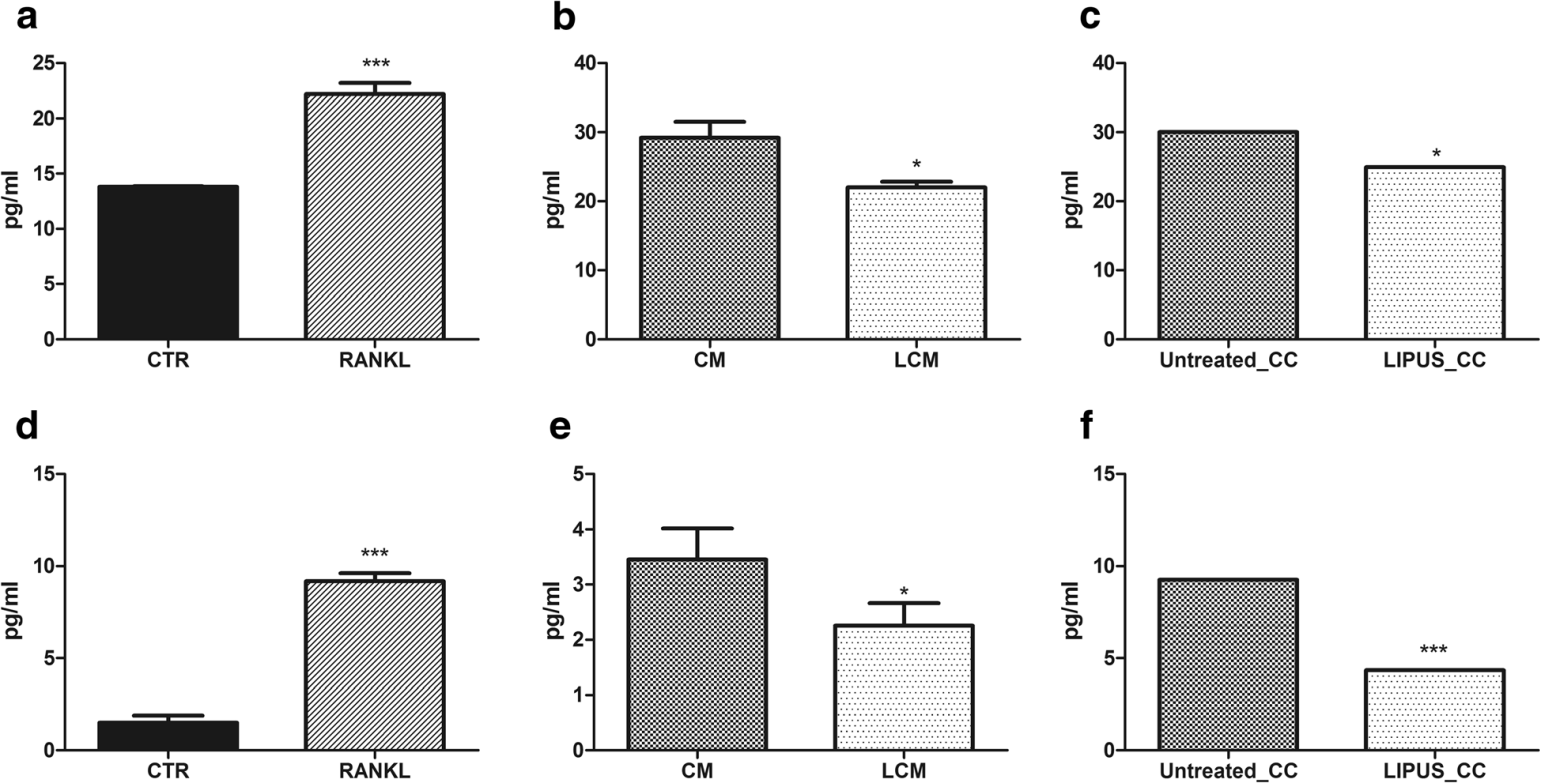
Osteclastic marker release. ELISA assay of CTSK (**a**-**c**) and MMP9 (**d**-**f**) were carried out onRaw264.7 cell cultures treated with: (**a**, **d**) RANKL; (**b**, **e**) conditioned medium harvested from MDA-MB-231 cell line untreated (CM) or treated (LCM) with LIPUS;or (**c**, **f**) co-cultured with MDA-MB-231 cells untreated (Untreated_CC) or treated (LIPUS_CC) with LIPUS, (Mean ± SD, *n* = 3 replicates). Student *t* test: *p < 0.05, **p < 0.005, ***p < 0.0005). Effect sizes between RANKL and CTR: CTSK (**a**) *d* = 11.71 and MMP9 (**d**) *d* = 18.3 for RANKL vs. CTR

**Fig. 5:**
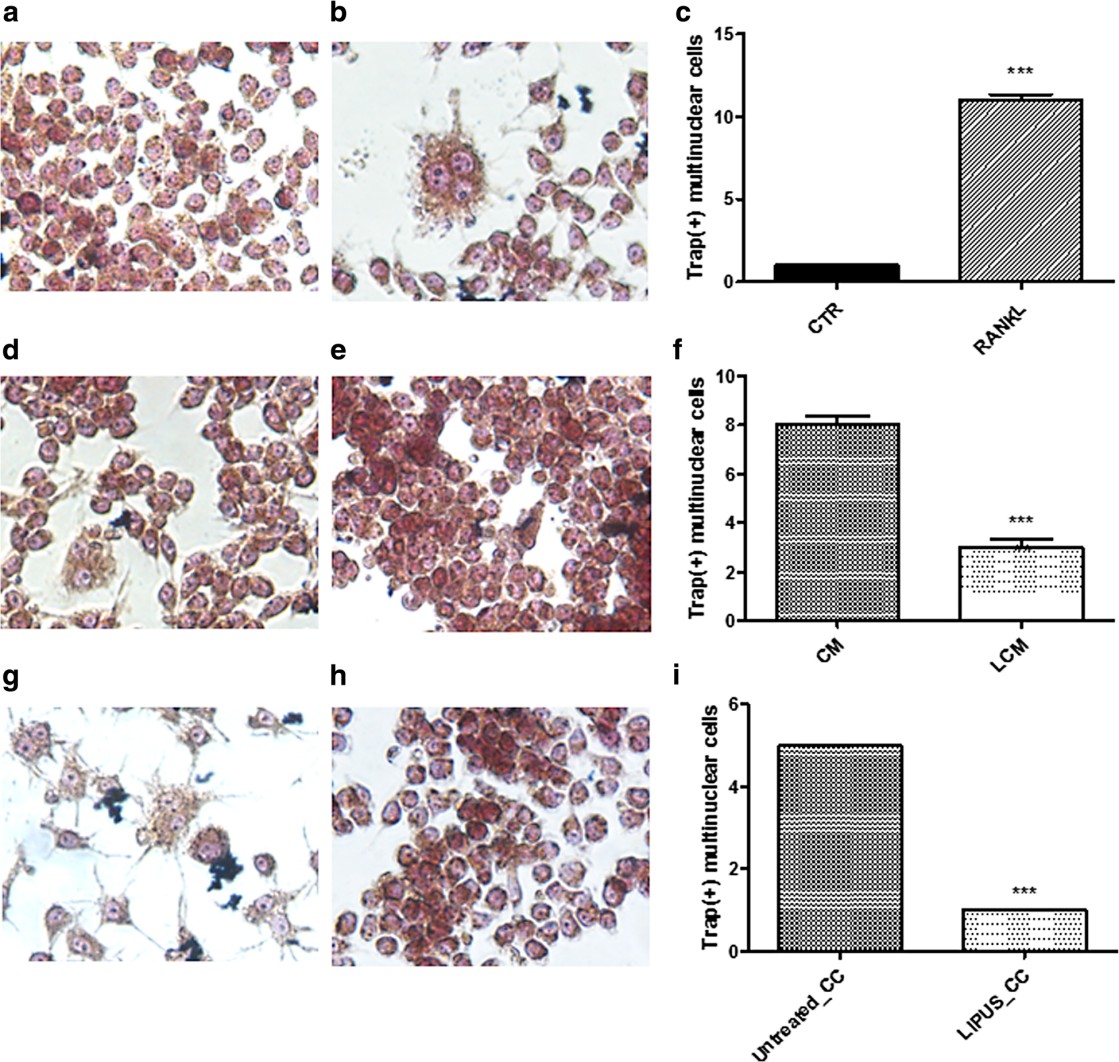
TRAP^+^ multinucleate cells. TRAP^+^ staining images (40X magnification) (**a**, **b**, **d**, **e**, **g**, **h**) and counts (**c**, **f**, **i**) performed on Raw264.7 cell cultures treated with: (**a**) CTR; (**b**) RANKL; conditioned medium harvested from MDA-MB-231 cell line (**d**) untreated (CM) or (**e**) treated (LCM) with LIPUS; or co-cultured with MDA-MB-231 cells (**g**) untreated (Untreated_CC) or (**h**) treated (LIPUS_CC) with LIPUS. TRAP^+^ cells were counted in three field: (**c**) RANKL vs. CTR; (**f**) LCM vs. CM. CTR; (**h**) LIPUS_CC vs. Untreated_CC, (Mean ± SD, *n* = 3 replicates). Student t test: **p < 0.005; ***p < 0.0005.Effect sizes between RANKL and CTR of TRAP+ cells: *d* = 41.6, *p* < 0.0005

**Fig. 6:**
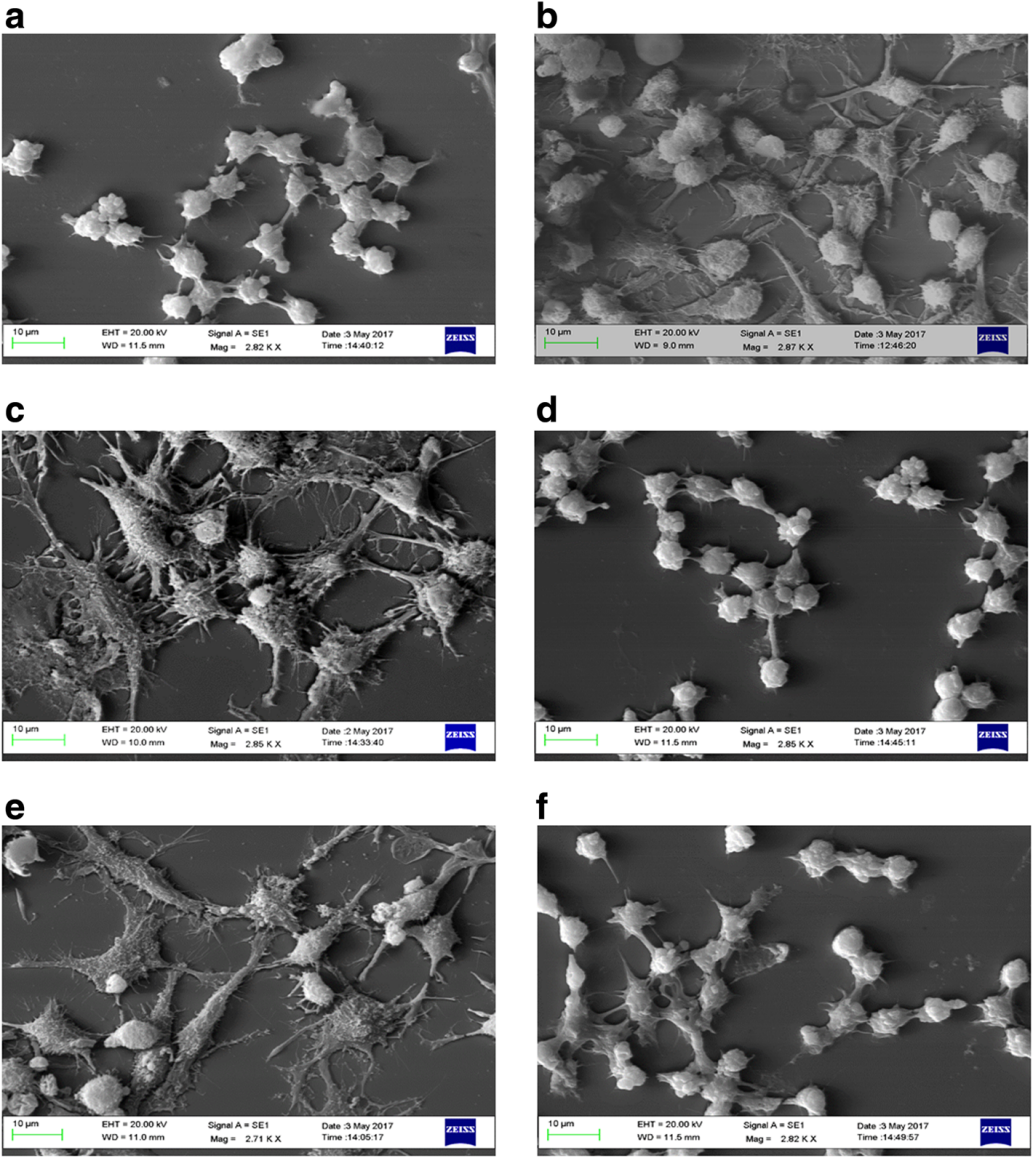
Ultrastructural analysis of Raw264.7 morphology. Panels are representative SEM imagesofRaw264.7 cell cultures treated with: (**a**) CTR; (**b**) RANKL; conditioned medium harvested from MDAMB231 cell line (**c**) untreated (CM) or (**d**) treated (LCM) with LIPUS; or co-cultured with MDAMB231 cells (**e**) untreated (Untreated_CC) or (**f**) treated (LIPUS_CC) with LIPUS

We assessed weather LIPUS treatment could influence MDA-MB-231 ability to induce osteoclastic differentiation in Raw264.7 cell line. To evaluate the system reliability, we cultured Raw264.7 cells in presence of RANKL treatment as positive control (Fig. 3a, d and g; Fig. 4a and d; Fig. 5b and c; and Fig. 6b).

Firstly, we evaluated the effects of LIPUS treatment on gene expression in Raw264.7 cells. Quantitative RT-PCR analysis showed an important gene expression modulation of osteoclast differentiation markers after LIPUS stimulation. In particular, in the indirect method cells treated with MDA-MB-231 conditioned medium obtained after LIPUS treatment (LCM) showed a significant decrease of *Ctsk* (*d =* − 2.6, *p* = 0.035) and *Mmp9* (*d* = − 3.0, *p* = 0.024) expression compared to cells treated with CM (Fig. 3e and h). In the direct method, LIPUS treatment on co-cultures (LIPUS_CC) caused a significant decrease of *Trap* (*d =* − 2.8, *p* = 0.026), *Ctsk* (*d =* − 2.3, *p* = 0.044) and *Mmp9* expression (*d* = − 2.8, *p* = 0.026) (Fig. 3c, f and i) compared to the untreated group (Untreated_CC), showing a general higher effect in co-culture system for *Trap* and *Ctsk* gene modulation.

Subsequently, we evaluated the effects of LIPUS treatment on proteins involved in bone resorption both by evaluating the release of CTSK and MMP9 and by assessing TRAP activity.

ELISA assays showed an important modulation of osteoclast differentiation markers release confirming gene expression data. In the indirect method, both CTSK and MMP9 release resulted decreased in the LCM group (*d* = − 4.2, *p* = 0.007 and *d* = − 2.4, *p* = 0.042, respectively) (Fig. 4b and e). In the direct method, LIPUS treatment caused a stronger decrease in CTSK and MMP9 release (*d* = − 4.2, *p* = 0.007 and *d* = − 10.8, *p* < 0.0005, respectively) than in the indirect method (Fig. 4c and f).

TRAP staining showed a significant reduction of multinucleate TRAP^+^ cells both in LCM (*d* = − 13.9, *p* < 0.0005) and LIPUS_CC group (*d* = − 11.0, *p* < 0.0005) (Fig. 5f, i) differently from CM and Untreated_CC groups.

These data show that LIPUS treatment is able to reduce the ability of MDA-MB-231 to induce osteoclastic differentiation of Raw264.7 cells in both systems.

### SEM analysis

Ultrastructural analysis demonstrated that a large number of Raw264.7 cells cultured in CM medium or in Untreated_CC (Fig. 6c and e) showed a characteristic osteoclastic morphology: cells appeared large, flat and with a ruffled border with numerous filopodia like in the RANKL treated group (Fig. 6b). On the contrary, the majority of cells cultured in LCM or in LIPUS_CC (Fig. 6d-f) showed amorphology similar between them and among undifferentiated Raw264.7, demonstrating that LIPUS treatment was able to reduce the ability of MDA-MB-231 to induce osteoclastic differentiation of Raw264.7 cells in both systems.

## Discussion

Ultrasound energy is a pressure wave that can produce both mechanical and thermal effects [27], which are not drastically separated [25] and whose applications are strongly dependent on intensity and frequency.

Ultrasound therapy can therefore be broadly divided into “low power” and “high power” applications, able to trigger a range of biological effects in relation to the exposure levels employed; although there is still no widely accepted definition of low-intensity ultrasound [44] the “low power” group includes physiotherapy and fracture repair (generally 30 mW/cm^2^, with 200 ms pulses and 1.5 MHz) [45], sonophoresis, sonoporation and gene therapy (usually 1.0–2.0 MHz at an intensity of 0.5 to 3.0 W/cm^2^) [44], whereas the most common use of “high power” ultrasound in medicine is high-intensity focused ultrasound (HIFU) (employed generally at an intensity≥3 W/cm^2^ and a frequency of 1–20 MHz) [27]. While useful therapeutic effects are now being demonstrated clinically, the mechanisms by which they happen are often not well understood [27]. The thermal effects of LIPUS are minimal, because of low intensity and pulsed output mode: the energy transported by an ultrasonic beam is attenuated passing through tissues, since it is scattered out elsewhere in the tissue. The ultrasound energy passing down into the tissues determines tissue molecular vibration, resulting in heat generation and tissue thermal changes [46]. The mechanical effects include acoustic streaming, secondary steady flow generated from the primary oscillatory [47], and stable cavitation, formation and growth of gas bubbles by accumulation of dissolved gas in the medium [46], which contribute mainly to the non-thermal effects in the application of LIPUS. The first mechanical effect can affect membrane permeability, diffusion rate and alteration of protein synthesis, cellular secretion, and sonoporation, while the second one improves drugs transport and cellular up-take [27].

LIPUS is a clinically established, widely used and FDA approved therapy to enhance bone growth during healing of non-union, fractures and other osseous defects [28, 48]. LIPUS induces mechanical stress in bone that, in turn, stimulates ossification of a soft callus through the modulation of calcium ion channels [49–52].

Conversely HIFU is already employed for tumor ablation [53, 54] and pain reduction [55] by causing tissue necrosis through the conversion of mechanical energy into heat (up to 80–90 °C within tissues) and unstable cavitation, which is the formation and immediate and violent collapse of gas-filled bubbles [26, 56, 57].

More recently, the use of LIPUS has been proposed in anticancer therapy due to its ability to increase the effects of anticancer drugs [58, 59]. LIPUS is able to modulate the expression of various soluble factors such as ILs and MMPs in both healthy and tumor cells [35-37] and breast cancer osteolytic capability depends on the release of these same soluble factors [12, 13]. Previously, Sawai et al. suggested that LIPUS used for bone metastases from renal and prostate cancer was able to induce osteoblastic differentiation without inducing cancer proliferation, vascularization, and migration [37]. The current study for the first time evaluated the effect of LIPUS on osteoclast precursors differentiation induced by osteolytic metastatic breast cancer cells, which were directly exposed to LIPUS to better mimic tumor niche.

We used a system consisting of metastatic breast cancer cells (MDA-MB-231) and murine macrophage cells (Raw264.7) [38, 60]. In particular, we evaluated osteoclastic morphology and activity by the expression and/or the release of typical markers: CTSK, a proteolytic enzyme responsible of bone resorption, MMP9, a gelatinase secreted by mature osteoclast involved in bone resorption and, finally, TRAP, a regulator of bone resorption through bone matrix degradation and highly expressed in terminally differentiated osteoclasts [61–63].

Our viability data showed no significant effects of LIPUS treatment on breast cancer cell lines; in particular, MDA-MB-231 viability was not altered after 10 days of LIPUS treatment similar to Sawai’s results about LIPUS effect on osteosarcoma and several others cancer cell lines. LIPUS treatment was able to decrease the capability of breast cancer cell culture medium to induce osteoclastic differentiation in Raw264.7, showing a significant reduction of multinucleate TRAP^+^ cells,as well as CTSK and MMP9 expression and release, using both direct and indirect approaches.

The current results suggested that the effect of LIPUS treatment on adopted in vitro microenvironment model determine the release of molecules, acting on osteoclast precursors limiting their differentiation. Previous studies about the directly effect of LIPUS treatment on osteoclasts alone showed contrastant results: Feres et al. [64] and Miyazaki et al. [65] showed an increase in osteoclast activity following treatment with LIPUS, while Monici et al. and Chen et al. [66, 67], showed a decrease in the expression of cytoskeletal components and markers of osteoclast growth, differentiation, and activity. In the tumor microenvironment, cancer cells coexist with preosteoclasts and release many soluble factors such as IL8, IL11, MMP1, MMP2, TNFα, and PGE_2_, which determine the direct osteolytic capability of breast cancer cells [12]. Interestingly, LIPUS treatment in co-culture system, which is closer to an in vivo setting, seemed to have a greater inhibitory effect on the osteolytic capacity of metastatic breast cancer cells, in particular for *Trap* and *Ctsk* expression and CTSK and MMP9 protein release. However, in our preliminary studies on IL8 and TNFα release by MDA-MB-231 treated and untreated with LIPUS, we did not find any association with osteoclast differentiation reduction (*data not shown*); therefore, we think that further investigations, such as proteomic profiling on MDA-MB-231 secretoma, might be useful to understand the pathways involved in this mechanism.

## Conclusions

In conclusion, our data suggest that LIPUS treatment is able to reduce in vitro osteolytic behavior of metastatic breast cancer cells. To overcome the current limit of this in vitro study, further investigations will be performed in 3D culture system in order to understand the reciprocal cellular cross-talk within the tumor microenvironment and the relative molecular basis involved [68]. Once the best treatment protocolsin the 3D system are identified, it will be possible to study LIPUS effects in vivo using a rat metastatic breast cancer model [69].

## Abbreviations

CM: Conditioned Medium
CTR: Control
CTSK: Cathepsin K
ELISA: Enzyme-Linked Immunosorbent Assay
FDA: Food and Drug Administration
FUS: Focused Ultrasound
GAPDH: Glyceraldehyde-3-Phosphate Dehydrogenase
HIFU: High Intensity Focused Ultrasound
IL: Interleukin
LCM: LIPUS Conditioned Medium
LIPUS: Low Intensity Pulsed Ultrasound
LIPUS_CC: LIPUS treated Co-cultures
MAPKs: Mitogen-Activated Protein Kinases
MMP: Matrix Metalloproteinase
PGE: Prostaglandine
PTHrP: Parathyroid hormone-related peptide
qRT-PCR: quantitative Real Time- Polymerase Chain Reaction
RANKL: Receptor activator of nuclear factor kappa-Β ligand
SATA: Spatial Average, Temporal Average
SD: Standard Deviation
SEM: Scanning Electron Microscopy
TNF: Tumor Necrosis Factor
TRAP: Tartrate Resistant Acid Phosphatase
Untreated_CC: Untreated Co-cultures
WST-1: [2-(4-Iodophenyl)-3-(4-nitrophenyl)- 5-(2,4-disulfophenyl)-2H-tetrazolium]
